# Monitoring work-related physical activity and estimating lower-limb loading: a proof-of-concept study

**DOI:** 10.1186/s12891-021-04409-z

**Published:** 2021-06-18

**Authors:** Xia Wang, Thomas A Perry, Jimmy Caroupapoullé, Alexander Forrester, Nigel K Arden, David J Hunter

**Affiliations:** 1 Department of Rheumatology, Royal North Shore Hospital, Institute of Bone and Joint Research, Kolling Institute, University of Sydney, 2065 St Leonards, Sydney, New South Wales Australia; 2 Centre for Sport, Exercise and Osteoarthritis Versus Arthritis, Nuffield Department of Orthopaedics, Rheumatology and Musculoskeletal Sciences, Botnar Research Centre, University of Oxford, Old Road, OX3 7LD Oxford, United Kingdom; 3 Faculty of Engineering and Physical Sciences, University of Southampton, Southampton, United Kingdom; 4 Independent Researcher, Town End Cottage, Grindon, Staffordshire, United Kingdom; 5 MRC Lifecourse Epidemiology Unit, Southampton General Hospital, University of Southampton, Southampton, United Kingdom

**Keywords:** Occupation, Physical activity (PA), Fitbit, Smartphone, Load-rate

## Abstract

**Background:**

Physical activity (PA) is important to general health and knee osteoarthritis (OA). Excessive workplace PA is an established risk factor for knee OA however, appropriate methods of measurement are unclear. There is a need to examine and assess the utility of new methods of measuring workplace PA and estimating knee load prior to application to large-scale, knee OA cohorts. Our aims, therefore, were to monitor workplace PA and estimate lower-limb loading across different occupations in health participants.

**Methods:**

Twenty-four healthy adults, currently working full-time in a single occupation (≥ 35 h/week) and free of musculoskeletal disease, comorbidity and had no history of lower-limb injury/surgery (past 12-months) were recruited across New South Wales (Australia). A convenience sample was recruited with occupations assigned to levels of workload; sedentary, light manual and heavy manual. Metrics of workplace PA including tasks performed (i.e., sitting), step-count and lower-limb loading were monitored over 10 working days using a daily survey, smartwatch, and a smartphone.

**Results:**

Participants of light manual occupations had the greatest between-person variations in mean lower-limb load (from 2 to 59 kg*m/s3). Lower-limb load for most participants of the light manual group was similar to a single participant in heavy manual work (30 kg*m/s3) and was at least three times greater than the sedentary group (2 kg*m/s3). The trends of workplace PA over working hours were largely consistent, per individual, but rare events of extreme loads were observed across all participants (up to 760 kg*m/s3).

**Conclusions:**

There are large interpersonal variations in metrics of workplace PA, particularly among light and heavy manual occupations. Our estimates of lower-limb loading were largely consistent with pre-conceived levels of physical demand. We present a new approach to monitoring PA and estimating lower-limb loading, which could be applied to future occupational studies of knee OA.

**Supplementary Information:**

The online version contains supplementary material available at 10.1186/s12891-021-04409-z.

## Introduction

Workplace and recreational/leisure physical activity (PA) has been shown to be a key contributor to maintaining and improving general health with low levels of PA associated with increased all-cause mortality [1, 2], particularly among those with pre-morbid conditions such as osteoarthritis (OA) [3]. Despite the known benefits of increasing levels of PA, increasing levels of work-related physical activity, as increasing workload, has been shown to carry an increased risk of disease incidence and progression [4–6]. Due to this, there has been a growing interest in measuring workplace PA.

Physical activity has been shown to play an important role in musculoskeletal disease, specifically OA [7–9]. Osteoarthritis, the most prevalent chronic joint disease, is a global health burden and is associated with pain, functional loss, and a reduction in quality of life. Despite evidence suggesting that PA, as ‘exercise’, with low levels of joint loading may benefit people with OA [10], excessive mechanical loading has been identified as a risk factor for disease [11, 12]. In the general working population, one of the major sources of repetitive and excessive knee force often comes from daily occupational activities. Many observational studies have found that manual occupations, such as construction work, mining [13] and farming [14–17], have a higher prevalence of knee OA compared to non-manual workers. Evidence has shown that many work-related physical activities such as heavy lifting, prolonged kneeling and squatting are associated with knee OA [18–20]. The underlying mechanism is thought to be related to the frequent exposure to knee loading, repetitive knee forces and a loss of cartilage lubrication, thus, resulting in joint structural damage [21–24].

Whilst work-related PA has been shown to be a risk factor for OA, appropriate methods for the measurement of workplace PA are however, unclear, and currently there are barriers to assessment. For instance, previous occupation-based observational studies [14–17] are often restricted to assessing occupational exposure(s) using self-reported surveys at a single visit; which is not reflective of long-term exposure patterns [25]. Further, estimating lower-limb load presents new challenges beyond measuring workplace PA and subsequently, biomechanical surrogate markers are frequently used including load rate; defined as the rate of change in load with respect to time [26, 27]. Knee loading can be evaluated using force plates in a controlled setting [28, 29] however, this method is often restrictive. More so, whilst other devices including inertial measurement units (IMUs) [30, 31] and smart-insoles [32] are available for the measurement of dynamic movements, these methods have not been used in real-world occupational settings.

The use of wearable devices (e.g., smartphones and smartwatches) to monitor aspects of rheumatology including symptoms, and daily workplace and leisure PA have become increasingly popular [33–37]. The use of smartphones and smartwatches to estimate and monitor the load rate on lower-limbs during recreational PA has been validated previously [38]. The load rates measured on the lower-limbs, using accelerometer data to estimate the load rate magnitude, has been shown to be highly correlated with the gold standard which measures ground reaction forces using force plates [38]. Whilst the framework used to estimate lower-limb loading has been pilot tested previously, in a single study in a real-world setting, and has demonstrated a sufficient degree of accuracy [38], the generalisability of these methods warrant further investigation.

More importantly, current methods used in occupational-based research to dichotomise occupations (i.e. non-manual and manual) or assign occupations to levels of workload (i.e. low, moderate and high) are largely based on overall physical exertion, such as step count and metabolic equivalents (METs), or focus on specific manual tasks i.e. predominately upper limb activities [39–41]. Such specific manual tasks are assumed to comprise the largest workplace PA component of the given occupation. Lower-limb load has rarely been taken into consideration when defining occupational levels, perhaps due to the complexities/restrictions of assessment and, very limited research exists to describe the loading on these joints. Studies are needed to evaluate lower-limb load and to determine whether current occupational classification systems adequately map lower-limb load to levels of workload.

In this study, our aims were: (i) to measure work-related PA and to estimate lower-limb load using a practical, real world approach using both self-reported surveys and commercially available smart devices across different occupations in healthy working-age adults and (ii) to classify levels of lower-limb loading in the workplace.

## Methods

### Study design

The current study was designed as a quantitative, exploratory, proof-of-concept prospective study. Participants’ work-related PA was monitored during working hours over a period of 10 working days. The study was approved by the Human Research Ethics Committee of the University of Sydney (Reference no 2019/846). All methods were performed in accordance with the relevant guidelines and regulations.

### Participants

A convenience sample of 24 participants were recruited from local communities in New South Wales (Australia). Eligible participants were aged 18 to 65 years, who were currently working for at least 35 h/week in a single occupation and were living in the greater Sydney area. Participants with a history of musculoskeletal disease, neurological or systemic illness, lower-limb injury and/or surgery during the last 12 months and/or co-morbid health conditions that could have prevented participation in the study (e.g., unstable angina, uncontrolled hypertension) and/or would impact on typical gait behaviour were excluded. Participants with a history of using walking aids and/or orthotic devices (i.e., crutches, knee braces and insoles) were also excluded as this could affect their lower-limb loading outside of the occupational exposure.

In line with previous job classification criteria, occupational job titles (e.g., office worker, farmer) were assigned to levels of workload including sedentary, light, light manual, and heavy manual groups [4, 42]; see Appendix 1.

### Study Procedure

All participants were asked to complete a daily, one-page diary survey via REDcap to record their daily working hours (start and end of the working day) and workplace physical activities. Participants were also asked to carry the smart devices (smartphone and Fitbit™) only during working hours. The samples of data recorded by smart devices were truncated according to the working hours reported in the daily surveys.

### Self-Reported Daily Survey

The daily surveys included compulsory fields for participants to complete. Self-reported intensity and the duration of workplace physical activities were collected. The daily survey was pre-populated with specific physical activities that were thought to be most common across the included occupations; these physical activities (e.g. walking, standing, lifting etc.) were informed by our previous work [6]. Participants also had the option to report other physical activities beyond those pre-populated in the survey. The start and finishing times for each working day, and work breaks, were recorded to allow the calculation of total working time. Participants’ daily physical activities during non-working hours (including commute time) were not measured.

### Smart Devices

PA metrics (i.e., tasks and step count) and lower-limb load estimates, described in more detail below, could not all be recorded using a single smart device. At the time of this study, the standard Fitbit™ report did not provide access to raw acceleration data which is essential for the estimation of lower-limb load. So, a Fitbit™ smartwatch was used to measure step count whilst a smartphone was used to capture raw accelerometer data to estimate lower-limb load rate.

Participants were provided with a smartwatch (Fitbit™ Versa series 2, Fitbit Inc., San Francisco, USA) and an Android smartphone (Samsung Galaxy™ A5, Samsung Electronics Inc., Suwon, South Korea), to carry in addition to their primary phone for 10 working days during working hours. Participants were asked to place the smartphone either in their trouser pocket or alternatively, strapped around their waist using a phone holder. Participants were also asked to wear the smartwatch on their non-dominant wrist which is a standard placement site for PA measurement using wrist-worn accelerometers as well as in compliance with the manufacturer recommendations [9, 31, 32].

### Smartwatch (Fitbit™ Versa series 2)

The increase of revenues generated by smartwatches [43] demonstrates that they are generally recognised in the global wearable market as the preferential way to monitor health and fitness levels. Fitbit™ smartwatches feature sensors designed for PA monitoring and have the capacity for on-board storage of approximately 7 days worth of data [44] without the need for syncing (offloading to an offsite server). Data captured by the Fitbit™ smartwatch was obtained via Bluetooth through the Fitbit™ mobile app. Fitbit™ uses proprietary algorithms [45] (not disclosed to the public) and provides estimates of step count at 60 s sampling intervals and daily estimates of physical activity.

### Smartphone (Samsung Galaxy™ A5)

The smartphone was used to monitor workplace physical activities and to capture raw accelerometer data which could be extracted specifically for each participant’s working hours. The smartphone was pre-installed with three smartphone applications (apps): (1) Fitbit™ app that was used to synchronise data captured by the smartwatch, (2) TouchTime™ [46] which is an app used for daily tracking of activity and (3) OApp™ to monitor and record estimates of load rates recorded by the phone [47].

#### a) Monitoring PA

The smartphone was preloaded with an app called TouchTime^TM^, which allowed participants to record the start and finishing time for a given activity during the working day. Participants were encouraged to record the time of each of their work breaks and non-wearing periods through the TouchTime™ app. In addition, within the app, participants were given an option to select one of eight predefined occupational activities (i.e., kneeling, lifting, carrying, climbing, squatting, walking, standing, and sitting) and use the TouchTime™ app to record the starting/finishing time when they were performing the given activity during the 10 working days. Within the app, participants had the choice to record other tasks/activities beyond those pre-listed and the frequency of the given physical activities (as counts).

#### b) Estimating Knee Load

Continuous logging of smartphone’s accelerometer data is not permitted by the power management restrictions of Android devices [48]. So, we chose to preload the smartphones with the OApp™ app which runs in the background of the phone unnoticed by the user, to capture raw accelerometer data [47]. The OApp™ app was developed in-house by researchers at the University of Southampton. OApp™ uses an intermittent random Monte-Carlo sampling of raw accelerometer data (with a sampling frequency of 50 Hz), which permits obtaining a statistical estimate of the load rate magnitude (load rate) and acceleration magnitude (acceleration) [38]. The estimated load rate is a surrogate measure for external impact load on the lower limbs using validated formulae as described in our previous study [47]. The infinitesimal calculus of the load rate is defined as:

$$\dot{\text{f}}=\frac{\text{d}\text{f}}{\text{d}\text{t}}=\text{m} \frac{\text{d}\text{a}}{\text{d}\text{t}}$$

The estimated mean load rate magnitude (kg m/s^3^) is defined as:

$$\widehat{\frac{\varDelta f}{\varDelta t}}= m \frac{ {\sum }_{i=1}^{i=n-1}\sqrt{{\left(\frac{{a}_{x,i+1}-{a}_{x,i}}{\varDelta t}\right)}^{2}+{\left(\frac{{a}_{y,i+1}-{a}_{y,i}}{\varDelta t}\right)}^{2}+{\left(\frac{{a}_{z,i+1}-{a}_{z,i}}{\varDelta t}\right)}^{2}}}{n-1}$$

where a_x_ = is the acceleration in x direction, a_y_ = is the acceleration in y direction, a_z_ = is the acceleration in z direction, n = the number of data samples at interval Δt (i.e. 1/sample frequency). OApp™ uses 5 s sampling windows (at 50 Hz) with 15 s intervals to estimate load rate as a surrogate for lower-limb load. Other load rate estimates, including accumulated load rate, variance and standard deviation were also calculated.

### Statistical analysis

Descriptive statistics (means and standard deviations) were calculated for each testing period and, for the mean of the steps/day and mean load rate/day. Data analyses were conducted using Python (version 3) and STATA (version 15.0). Summary statistics for the Fitbit™ were provided on a 24-hour cycle, whilst data from the smartphone could be extracted on a per working-hour basis.

## Results

### Participant demographics

A total of 24 healthy participants were recruited and completed the study; see Table 1. Five, eighteen and one study participant were classified as working in sedentary, light manual and heavy manual occupations, respectively. No participants were recruited from light occupations.

**Table 1:** Baseline characteristics of participants by workload levels

	Workload			Total (*N* = 24)
	**Sedentary** **(***N*** = 5)**	**Light Manual ** **(***N*** = 18)**	**Heavy Manual ** **(***N*** = 1)**	
**Age (years), mean (SD)**	33 (5.8)	42.4 (12.6)	30 (0)	39.9 (11.9)
**Gender, female n (%)**	2 (40.0)	3 (16.7)	0 (0)	5 (20.8)
**BMI (kg/m**^**2**^**), mean (SD)**	22.6 (3.1)	27.6 (6.9)	26.9 (0)	26.6 (6.4)
**Ethnicity**				
Caucasian	1 (20.0)	11 (61.1)	1 (100)	13 (54.2)
Asian	3 (60.0)	6 (33.3)	0 (0)	9 (37.5)
Other	1 (20.0)	1 (5.6)	0 (0)	2 (8.3)
**Education**				
High school or less	0 (0)	10 (55.5)	0 (0)	10 (41.7)
Apprenticeship/vocational school	0 (0)	3 (16.7)	0 (0)	3 (12.5)
University degree or higher	5 (100)	5 (27.8)	1 (100)	11 (45.8)
**Living status**				
House	0 (0)	11 (61.1)	0 (0)	11 (45.8)
Apartment	5 (100)	7 (38.9)	1 (100)	13 (54.2)
**Relationship status**				
Single	1 (20.0)	5 (27.8)	1 (100)	7 (29.2)
Married/living with a partner	4 (80.0)	12 (66.7)	0 (0)	16 (66.7)
Not disclosed	0 (0)	1 (5.5)	0 (0)	1 (4.2)
**Annual income (Australian dollars)**				
<$50,000	1 (20.0)	3 (16.7)	0 (0)	4 (16.7)
$50,000–$99,999	1 (20.0)	10 (55.5)	0 (0)	11 (45.8)
>$99,999	2 (40.0)	3 (16.7)	0 (0)	5 (20.8)
Not disclosed	1 (20.0)	2 (11.1)	1 (100)	4 (16.7)
**Employment status**				
Corporate/government employee	2 (40.0)	11 (61.1)	0 (0)	13 (54.2)
Contractor or self-employed	0 (0)	4 (22.2)	1 (100)	5 (20.8)
Others	3 (60.0)	3 (16.7)	0 (0)	6 (25.0)
**Weekly working hours, mean (SD)**	42.5 (5.3)	43.4 (7.3)	35.0 (0)	42.9 (6.9)
**Years working in current job**, **mean (SD)**	2.1 (0.91)	11.2 (11.3)	0.5 (0)	8.9 (10.6)
**Age started working (years), mean (SD)**	25.8 (1.9)	18.7 (4.0)	29 (0)	20.6 (4.9)
**Exercises during workday, yes**^**≠**^	1 (20.0)	3 (16.7)	0 (0)	4 (16.7)
**Previous joint injury at work, yes**	0 (0)	4 (22.2)	0 (0)	4 (16.7)
**How would you classify your level of physical demand in your current job?**				
Sedentary^1^	5 (100)	0 (0)	0 (0)	5 (20.8)
Light to moderate^2^	0 (0)	14 (77.8)	1 (100)	15 (62.5)
Intensive^3^	0 (0)	4 (22.2)	0 (0)	4 (16.7)

Data presented as counts (percentage) unless stated otherwise.

Abbreviations: *BMI* body mass index, *SD* standard deviation.

† ‘Other’ category includes mixed ethnicities.

**≠**Positive response to: ‘In a typical working day, did you do any vigorous sports or exercise (including cycling/running to work) for more than 30 minutes?

^1^Sitting, desk work or very light manual tasks.

^2^Walking, standing, light tool or machinery work.

^3^ Heavy tool or machinery work, heavy manual labour.

### Workplace physical activity (self-assessed via daily surveys)

80 % (19/24) of participants completed the self-reported, daily surveys for 10 days, 4 participants completed at least 7 days and one participant completed 6 days; the number of completed surveys corresponds to the number of days the respective participants stayed in the study. The number of hours spent at work per day was 8.93 (±2.04 h), including approximately a one-hour break (Table 2). The TouchTime™ app was provided as a way to record PA ad-hoc but none of the participants reported PA beyond completing the daily surveys.

**Table 2:** Comparisons of daily work activities performed by workload levels; data acquired using daily surveys

	Workload			Total (*N* = 24)
	**Sedentary** **(***N*** = 5)**	**Light Manual ** **(***N*** = 18)**	**Heavy Manual** **(***N*** = 1)**	
**Working hour**^**≠**^ (hours)	8.0 (1.45)	9.33 (2.04)	6.65 (2.07)	8.93 (2.04)
**Work break** (hours)	0.93 (0.45)	1.05 (0.58)	0.64 (0.24)	1.01 (0.55)
**Sitting** (hours)	6.58 (1.07)	1.90 (1.32)	0.72 (1.09)	2.87 (2.34)
**Standing** (hours)	0.53 (0.56)	2.62 (2.18)	4.5 (2.25)	2.24 (2.17)
**Walking** (hours)	0.61 (0.48)	3.83 (2.30)	3.06 (2.27)	3.01 (2.43)
**Kneeling** (hours)	0.38 (0.18)	0.64 (0.43)	2 (0)	0.65 (0.45)
**Squatting** (hours)	0.05 (0.05)	0.51 (0.38)	1.17 (0.68)	0.54 (0.44)
**Lifting** (kg)	0 (0)	7.29 (7.28)	10.75 (13.07)	7.42 (7.50)
**Lifting** (times)	0 (0)	123 (250.0)	41 (40.34)	120 (245.83)
**Carrying** (kg)	0.5 (0)	7.38 (5.93)	14 (13.89)	7.53 (6.35)
**Carrying** (times)	0 (0)	129 (291.7)	7 (2.89)	124 (286.08)
**Climbing** (number of stairs)	43 (56)	72 (145)	5 (0)	67 (136)

Data presented as means and standard deviation (SD).

All results presented here correspond to the physical activities performed during working hours only.

Participants classified as working in sedentary occupations reported the longest hours of sitting (6.58±1.07 h/day), participants of the heavy manual group reported the longest time spent kneeling (2±0 h/day), squatting (1.17±0.68 h/day) and standing (4.5±2.25 h/day) whilst the light manual group reported the greatest time spent walking (3.83±2.30 h/day), number of stairs climbed (72±145/day) and, greatest frequency of lifting (123±250.0/day) and carrying (129±291.7/day). In addition, the heavy manual workload group reported lifting the heaviest items and, carrying the heaviest goods more frequently than the other two groups (Table 2).

### Estimation of step count (via Fitbit)

**Fig. 1:**
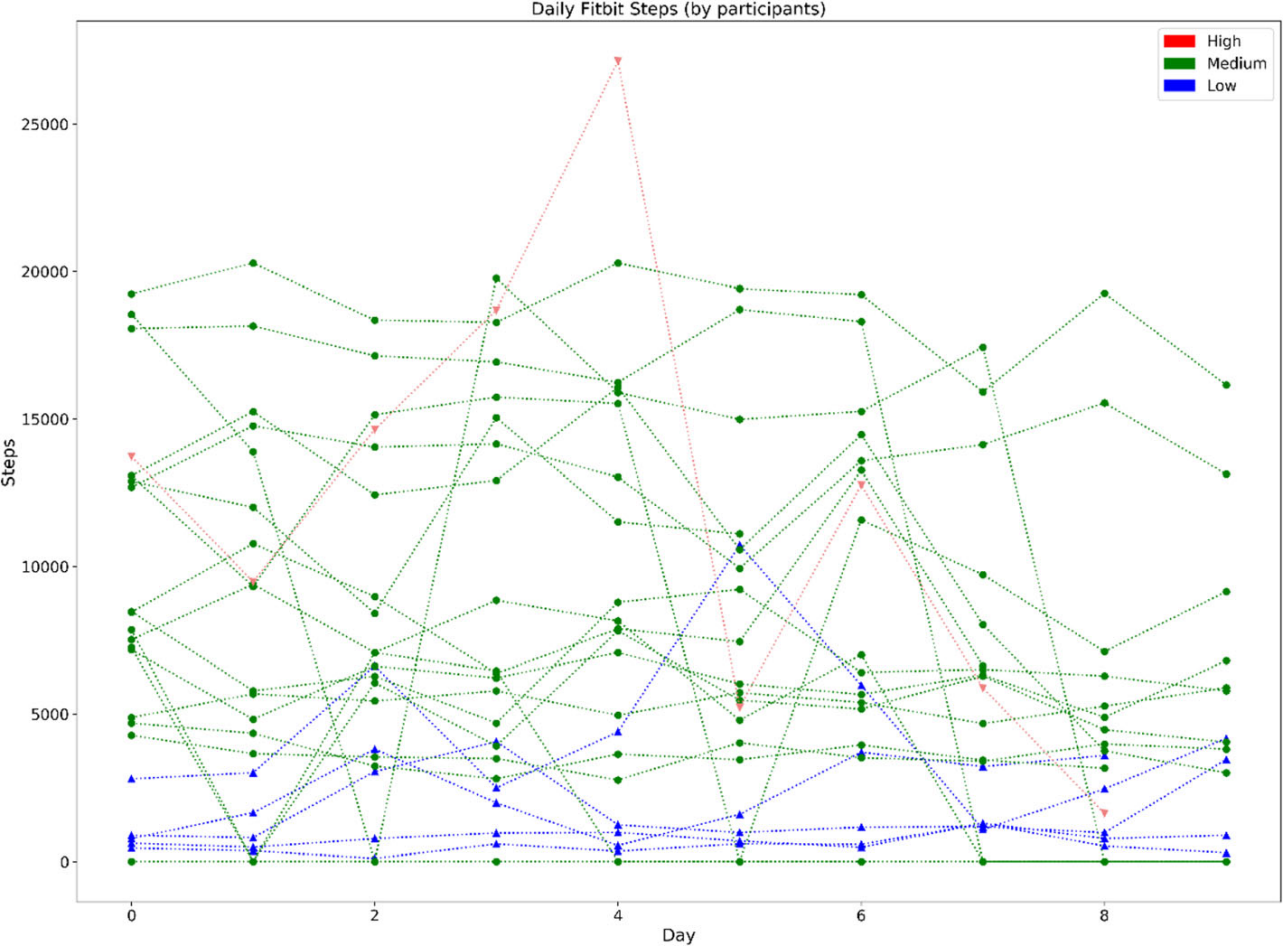
Description of physical activity parameters captured by Fitbit™ during working hours over 10 working days grouped by workload levels. Each data point corresponds to the activity measure per day with each line corresponding to a single study participant. Participants are grouped by workload: blue = sedentary, green = light manual and red = heavy manual.

Participant step counts were exported using Fitbit’s report and then truncated to working hours in accordance with the participants’ responses to the daily surveys. There were missing data for two participants due to smartphone issues (e.g., data synching and device handling). 95 % (23/24) of participants wore the Fitbit™ smartwatch with 17 participants completing 10 days and 5 participants completing at least 7 days. Figure 1 shows the daily step count captured by the smartwatch for each respective workload.

When looking at daily step count across the respective workloads, participants of the sedentary group showed the most stable (i.e., linear) individual trends in step count over time; with all sedentary participants demonstrating similar trends. The greatest between-person variations and fluctuations over time were observed among participants of the light manual group; with some participants demonstrating at least three times more daily steps compared to the participants of the light manual group with the fewest steps (see Fig. 1). Whilst based on a single study participant, we found that the heavy manual worker had more than twice the daily average step count compared to sedentary workers (see Fig. 1). The heavy manual worker showed variations in step count over time but overall, most participants’ individual trends were largely consistent with small fluctuations over time. Similar trends were observed across each individual job title (see Appendix 2).

### Estimation of Lower-limb loading

Participant-specific working hours were determined using the responses to the daily survey, with 17 participants completing 10 days, 4 participants completing 8 days and one participant completing 6 days. Additionally, there were missing Fitbit™ data for two participants.

**Fig. 2:**
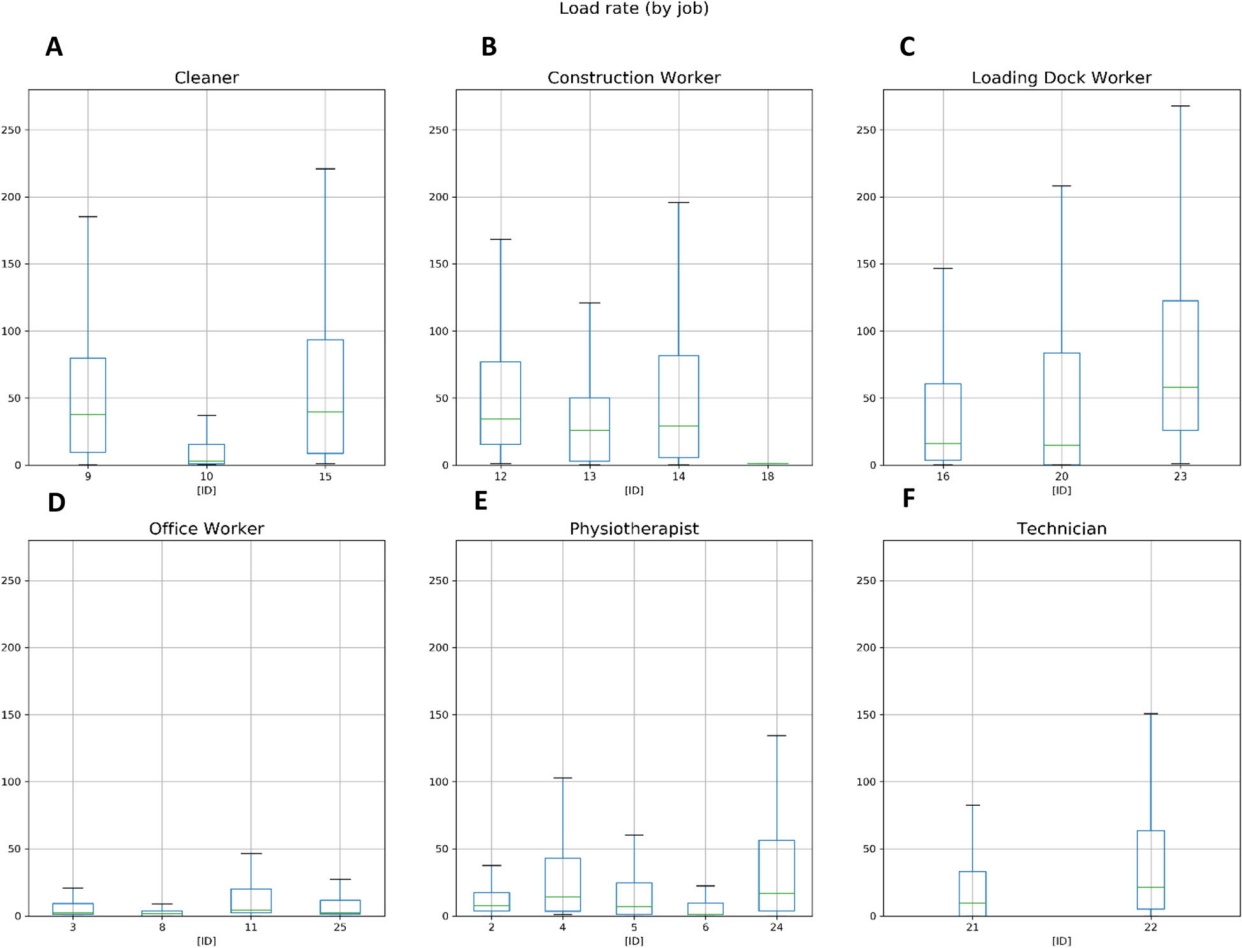
Estimated lower-limb load, captured by the smartphone, during working hours over 10 working days of all participants grouped by job categories. The boxplots show the median (green line), first and third quartiles (box), min and max (whiskers) excluding outliers of the estimated lower-limb loads (kg*m/s3) for each respective study participant grouped by job category; (**A**) cleaner (*N* = 3), (**B**) construction worker (*N* = 4), (**C**) loading dock worker (*N* = 3), (**D**) office worker (*N* = 4), (**E**) physiotherapist/occupational therapist (*N* = 5) and (**F**) technician (*N* = 2)

Over the 10-day period, we observed that lower-limb load within occupational categories (e.g., cleaner, construction worker) was not evenly distributed. Figure 2 shows the estimates of daily mean lower-limb loading (kg*m/s3) for each study participant across the six occupational categories. Loading dock personnel had the greatest mean daily lower-limb load, followed by construction workers and cleaners. In contrast, compared to loading dock personnel/construction workers, the mean load rate for office workers was three times smaller. The daily mean lower-limb load for physiotherapists/occupational therapists and technicians was greater than office workers.

When looking at the load rate within workload levels, we observed that the intra-participant variations within heavy and light manual groups was at least three times greater than for sedentary occupations (i.e., office workers).

**Fig. 3:**
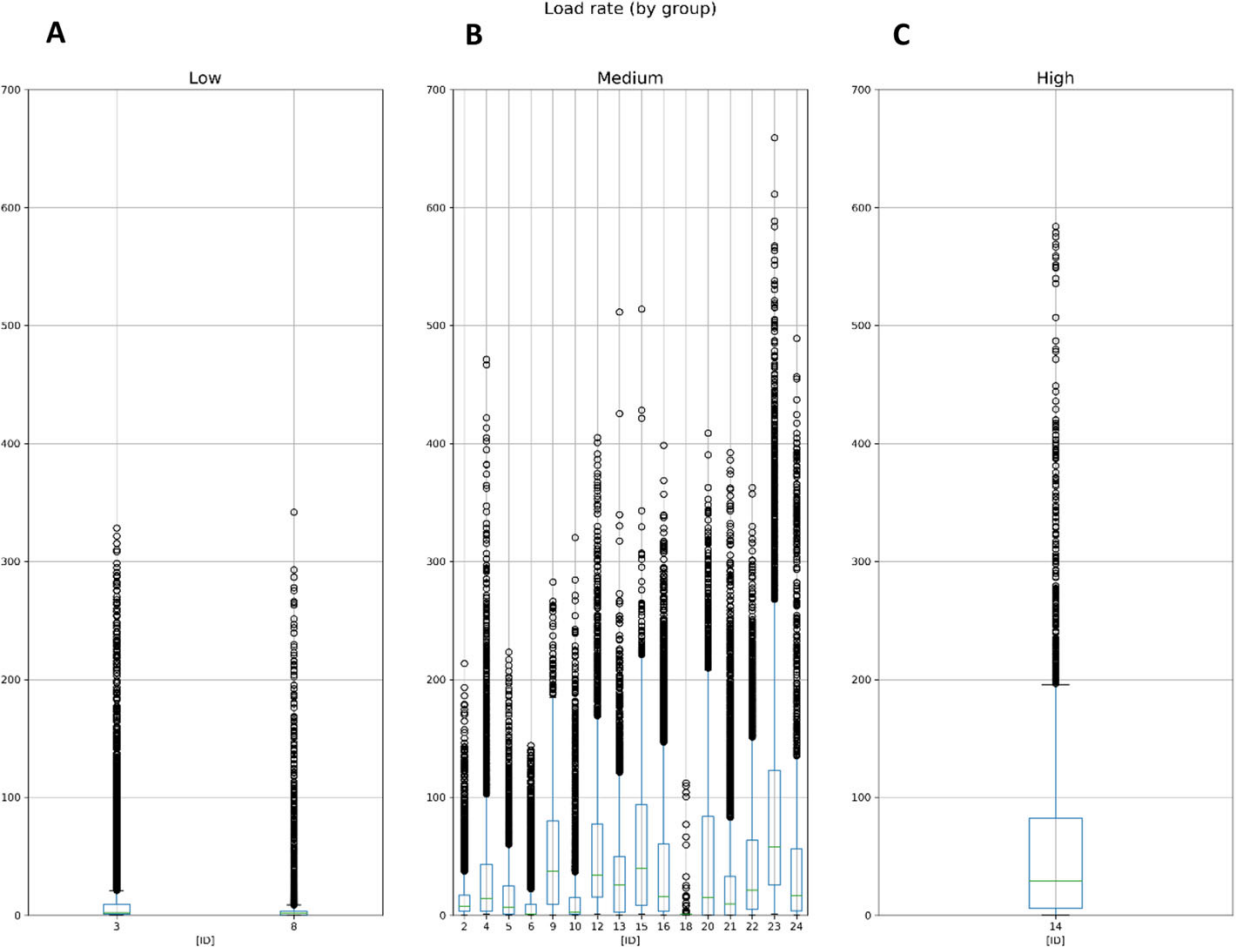
Estimates of lower-limb load by workload levels. The boxplots show the median (green line), first and third quartiles (box), min and max (whiskers) including outliers of the estimated lower-limb loads (kg*m/s3) for each respective study participant grouped by workload levels; (**A**) sedentary (*N* = 2), (**B**) light manual (*N* = 16) and (**C**) heavy manual (*N* = 1).

Figure 3 shows the agglomerated lower-limb load (kg*m/s3) data for each study participant across levels of workload (i.e., sedentary, light manual and heavy manual). The dispersion in lower-limb load was more significant for some workloads than others. The outliers shown here indicate load rates that far exceeded the median and standard deviation values; these were typically ten times greater than the median lower-limb load for each participant.

## Discussion

This study is the first to monitor and compare workplace PA in a real-world setting and to estimate loading of the lower limbs across different occupations using self-reported surveys and smart devices. Our key findings were that monitoring of PA using smart devices provides insights, further to self-reported assessments, that could help evaluate load variations on the lower limbs. Most participants classified as light manual, using pre-existing definitions, were found to demonstrate a range of loads that were similar to a single participant in heavy manual work. Rare events of extremes in lower-limb loading demonstrated by all participants poses the question of whether the frequency and intensity of such extreme lower-limb loads contributes to OA disease.

Our key findings were that participants classified, using pre-existing definitions, as working in light manual jobs, including physiotherapists and technicians, reported more intensive lower limb activity such as frequency of lifting and number of stairs climbed compared to sedentary and heavy manual occupations. Further, participants of this group showed the greatest variation in step count and load rate patterns over time compared to other groups. This would suggest that our estimates of lower-limb load and step count are sensitive to such activities involving the lower limbs. Furthermore, whilst limited to a single study participant, a heavy manual worker (i.e., construction labourers) had a greater step count and mean daily load rate compared to light manual and sedentary occupations. More so, the heavy manual worker reported greater time spent performing manual tasks (i.e., lifting) than light manual and sedentary occupations, respectively.

Our study improves the current understanding of workplace PA by identifying patterns of routinely performed physical activities and estimated lower-limb loading. These data suggest that current methods used to assign occupations to levels of activity, which are mostly informed by whole-body activity measures and/or assumed work tasks, may not be applicable for large scale application given the vast within-occupation variations in workplace activities and, the levels used may not be reflective of loading of the lower limbs. For instance, the greatest variation in daily mean load rate occurred within participants working in light manual occupations whilst the behaviours of workers in sedentary occupations were more stable over time. These data also go some way to describing patterns of PA which may help to identify the underlying pathologies of knee OA. For instance, it is highly contested as to whether OA is driven solely by duration, frequency or intensity of lower-limb loading; it is likely to be a combination of these factors with data supporting that both intensity and duration of daily-living PA influence risk of disability in OA [49]. Construction work has been routinely shown to carry an increased risk of knee OA due to an excessive level of knee loading. Similarly to the light manual group, we showed in a single construction worker that loading of the lower limbs fluctuated considerably on a daily basis; thus, it could be these harsh fluctuations in lower limb loading across both light and heavy manual occupations that may be a driving factor for OA. Further research is needed to take into account measures of lower limb movements. The previously validated load rate magnitude algorithm [47], which was built into the OApp™, has been tested beyond previous studies and has shown a great potential to monitor lower limb loading in a real-world environment using commercially available smart devices.

There are, however, many challenges to using smart devices to monitor workplace activity in a real-world setting. Obstacles for use include workplace restrictions, hazardous working environments and/or interference with existing professional equipment. Our study found that compliance varied among occupational groups as demonstrated by the varying completion rates of the 10-day wearing time. We found that the single construction worker often failed to wear the smart device as instructed for the 10-day period, particularly the smartphone. Reasons for poor compliance, as reported by the study participants included workplace restrictions or the inconvenience caused by wearing extra devices during heavy physical work. In addition, the unstable/irregular working schedules and impact from weather or other environmental changes were also potential contributors. In our study, levels of compliance for wearing the Fitbit™ and/or smartphone were comparable to previous studies of wearable devices in participants with knee OA [34].

Technical issues are also potential barriers to data capture, which need to be considered to facilitate the wider use of smart devices in future practice. In our study, the smartphones were used as secondary devices which led to issues uploading data (Fitbit™ and OApp™ app) and devices entering ‘doze mode’ during extended periods of inactivity [50] (e.g. participants not interacting regularly with the device). Despite all participants reporting use of the smartphone for the duration of the time spent in the study, load rate estimates were missing for 4 study participants and 10 participants had load rate estimates missing for 1 to 4 days. This resulted in a total of 20 participants with at least 6 days of data for the analyses.

Participants filled the daily survey required for the study but did not make use of the timer (Touchtime™ app) to record activities during work, which indicates that ad-hoc self-reported assessments used to report PA might not apply in real life. The start and end of working hours could only be determined by self-reported assessment (as opposed to using AI/ML techniques) and so this study demonstrates that self-reported surveys and smart devices cannot be used in isolation in order to monitor workplace PA and estimate lower-limb loading.

There are several limitations to this study which require careful consideration. Firstly, our study population was small (N = 24). As this was an explanatory, real-world setting, proof-of-concept study, a standard sample size calculation was not required. By taking a convenience sample approach, we were able to recruit participants across 3 of the 4 workload levels (sedentary, light manual and heavy manual) [4]. Ideally, we would have recruited more participants from ‘light’ and ‘heavy manual’ groups in order to facilitate a more robust assessment of between-participant comparison. As our findings for heavy manual work are based on a single study participant, our results should be interpreted with caution and require further validation in a larger study sample. Our ability to recruit only a single study participant from heavy manual occupations is an important finding in itself. It demonstrates the real-world issues of recruiting for wearable-based studies, from a healthy population, and it highlights that it may be more difficult to recruit from certain occupational sectors. This should help to inform the recruitment strategies of future wearable-based occupational studies as efforts may need to be targeted and localised to specific working groups. The study has a relatively short follow-up period however, we were able to capture shift-patterns and those that were likely to fluctuate over a 2-week period. We do, however, acknowledge that some of the included job titles may work to a shift pattern beyond the two weeks measured here. A further limitation of this study was that we did not correlate the level of physical activity (e.g., step count) as measured by wearable devices to patient-reported questionnaires.

Whilst it has been reported that the use of force plates in a gait lab setting is the most accurate method of determining lower limb loading, the limitations of this approach are well known and are restricted to a non-real world setting. Our approach to determining estimates of lower-limb loading is more practical and was conducted in real world, occupational settings. We asked the study participants to carry a secondary phone, rather than loading the device applications onto their primary phone. The limitations to using a secondary device are well known [50]. However, this was considered the most appropriate way of measuring physical activity measures during working hours. Due to power management of the smartphone [51] it is not possible to continuously record raw sensor data and so, we conducted a sample-based analysis. Finally, we asked patients to self-report the start and end of each working day to allow estimation of working hours; this is likely to be subject to recall bias, however, this was the most feasible and commonly used approach to acquire such data.

## Conclusions

Our findings suggest that smart devices are feasible for monitoring workplace physical activity and for estimating lower limb loading in most occupational settings. Our observations of step count with new estimates of lower-limb load during work were largely consistent with pre-defined levels of physical demand. There were large interpersonal variations in light manual and heavy manual workers and also large fluctuations in their workload/shift patterns. We present a new approach to monitoring workplace physical activity and estimating lower-limb load utilising daily survey data and smart devices which could be applied to future occupational studies.

## Supplementary Information

**Additional file 1:**

**Additional file 2:**

## Data Availability

The datasets generated during and analyzed during the current study are not publicly available due to data protection purposes but are available from the corresponding author on reasonable request.
