# Supplementary material for: Monitoring work-related physical activity and estimating lower-limb loading: a proof-of-concept study
Source: BMC Musculoskelet Disord. 2021 Jun 18;22:552. doi: 10.1186/s12891-021-04409-z (PMC8212530; doi:10.1186/s12891-021-04409-z)
Supplement: Supplementary file 1 — Additional file 1: [file 12891_2021_4409_MOESM1_ESM.docx]

**Appendix 1:** Categorisation of job titles according to levels of physical demand for included study participants.

| **Workload Level** | **Occupational Task(s)** | **Job Title(s)** | **Number of participants** |
| --- | --- | --- | --- |
| Sedentary | Predominantly sitting | Office workers | 5 |
| Light | Sitting or standing with some walking | - | 0 |
| Light Manual | Walking, with some handling of materials weighing <50 pounds | Physio/occupational therapist | 5 |
|  |  | Cleaner | 4 |
|  |  | Plasterboard Installer | 2 |
|  |  | Loading dock worker | 4 |
|  |  | Technician | 2 |
|  |  | Painter | 1 |
| Heavy Manual | Walking, with frequent handling of materials weighing >50 pounds | Construction workers (general labour) | 1 |
