# Supplementary material for: Monitoring work-related physical activity and estimating lower-limb loading: a proof-of-concept study
Source: BMC Musculoskelet Disord. 2021 Jun 18;22:552. doi: 10.1186/s12891-021-04409-z (PMC8212530; doi:10.1186/s12891-021-04409-z)
Supplement: Supplementary file 2 — Additional file 2: [file 12891_2021_4409_MOESM2_ESM.docx]

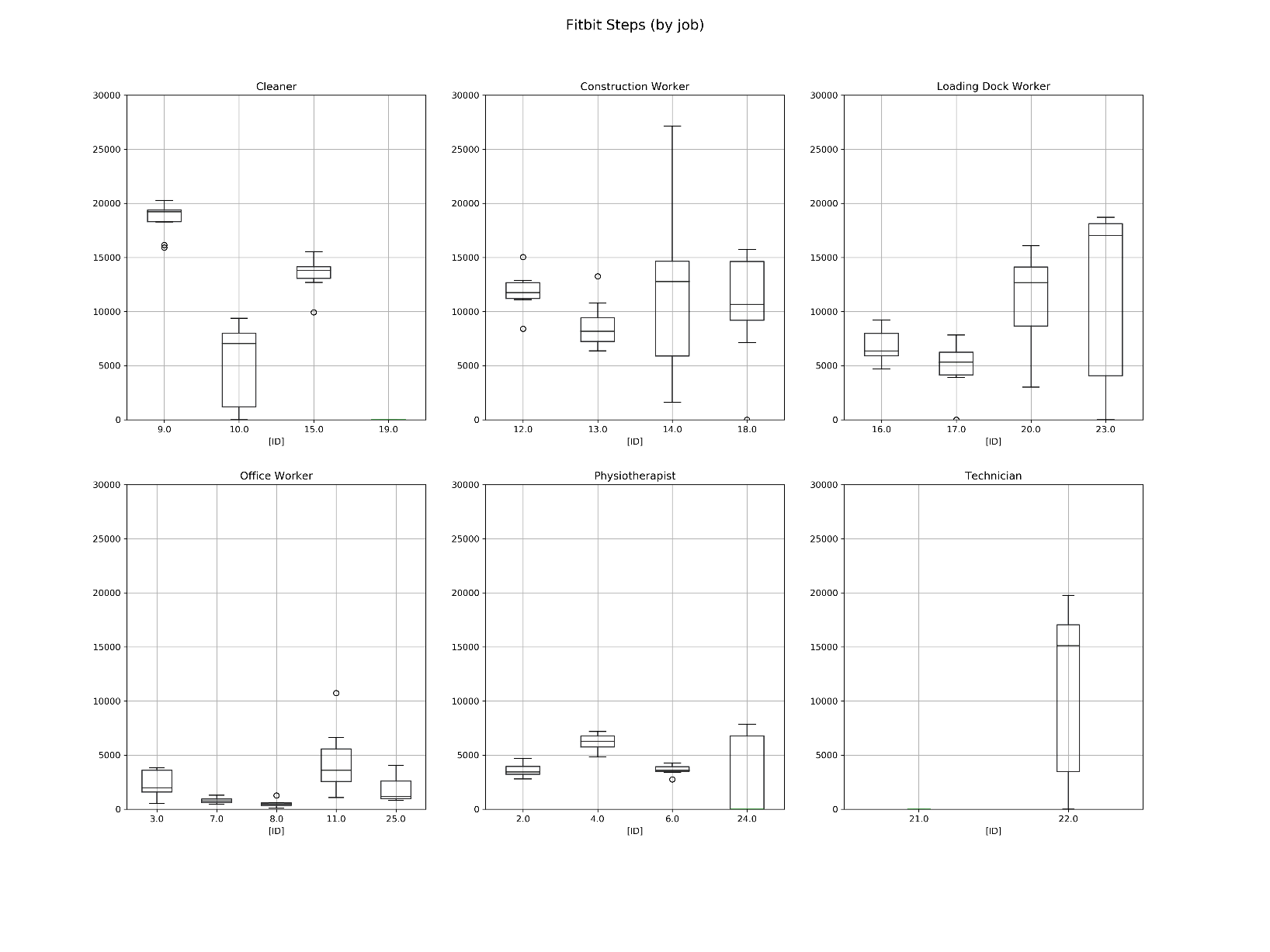


**A**

**B**

**C**

**D**

**E**

**F**

**Appendix 2: Description of daily step counts captured by Fitbit^TM^ during working hours over 10 working days grouped by occupational title**. The boxplots show the median (green line), first and third quartiles (box), min and max (whiskers) excluding outliers for each respective study participant grouped by occupational title; (A) cleaner (N = 4), (B) construction worker (N = 4), (C) loading dock worker (N = 4), office worker (N = 5), physiotherapist/occupational work (N = 4) and technician (N = 2).
